# Evaluation of blood glucose control in ICU patients with Space GlucoseControl: a European study

**DOI:** 10.1186/cc13628

**Published:** 2014-03-17

**Authors:** J Blaha

**Affiliations:** 1 General University Hospital, Prague, Czech Republic

## Introduction

Regardless of the ongoing debate on optimum target ranges, glycaemia control (GC) remains an important therapeutic goal in critically ill patients. Dozens of different insulin protocols for ICUs have been developed with different complexity, effectiveness, blood glucose (BG) variability and safety. Although comparison of existing protocols is difficult due to significant differences in processes and outcome measures, computerized clinical decision support systems generally achieved better GC with consistently lower hypoglycaemia rates than that achieved with paper-based protocols [1]. The enhanced Model Predictive Control (eMPC) algorithm, developed by the CLINICIP group, is the effective clinically proven protocol, which has been successfully tested at multiple institutions on medical and surgical patients with different nutritional protocols [2,3]. The eMPC models the behaviour of glucose and insulin in ICU patients with a variable sample interval based on the accuracy of the BG prediction. It has been integrated in the B.Braun Space GlucoseControl (SGC) system, which allows direct data communication between pumps and Space Control with the incorporated eMPC algorithm. Although SGC is already clinically used in dozens of ICUs worldwide, there are few only published experiences with its use [4].

## Methods

The primary objective of this multicentre European noninterventional study was to evaluate the performance (efficiency) of SGC under routine conditions in adult ICU patients requiring BG control. The primary endpoint was the percentage of time within the target range, and secondary outcome measures were the frequency of hypoglycaemic episodes and BG measurement intervals. Patients in this trial were assigned to the target range 4.4 to 8.3 mmol/l. Nutritional management (enteral, parenteral or both) was carried out at the discretion of the each centre.

## Results

Seventeen centres from nine European countries included a total of 508 patients. During the study a total of 29,575 BG values were entered into the SGCs and the same number of recommendations were rendered. The mean time-in-target was 77.5 ± 20.9%. The mean proposed next measurement time was 2.0 ± 0.5 hours. Only four episodes of hypoglycaemia <2.2 mmol/l occurred (0.01% of measurements).

## Conclusion

SGC is a safe and very efficient system to control BG in ICU patients.

## References

[B1] Jacobi J Crit Care Med 2012 40 3251 3276 10.1097/CCM.0b013e3182653269 23164767

[B2] Cordingley JJ Intensive Care Med 2009 35 123 128 10.1007/s00134-008-1236-z 18661120

[B3] Blaha J Diab Care 2009 32 757 761 10.2337/dc08-1851 PMC2671097 19196894

[B4] Amrein K Diabetes Technol Ther 2012 14 690 695 10.1089/dia.2012.0021 22694176

